# Supplementary material for: Cell-Type-Dependent Recruitment Dynamics of FUS Protein at Laser-Induced DNA Damage Sites
Source: Int J Mol Sci. 2024 Mar 20;25(6):3526. doi: 10.3390/ijms25063526 (PMC10971167; doi:10.3390/ijms25063526)
Supplement: Supplementary file 1 [file ijms-25-03526-s001.zip › ijms-2879190-supplementary.pdf]

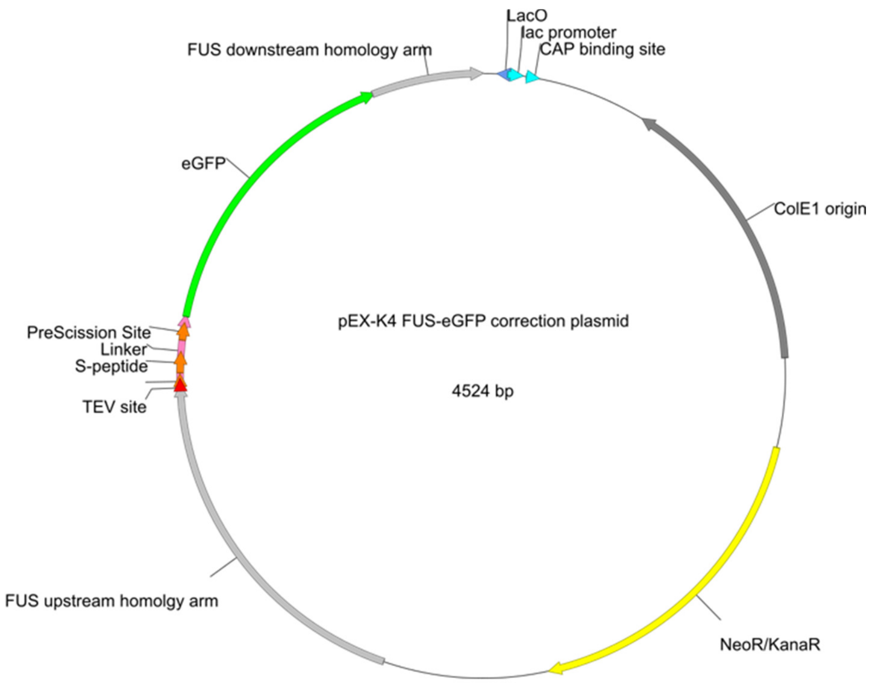

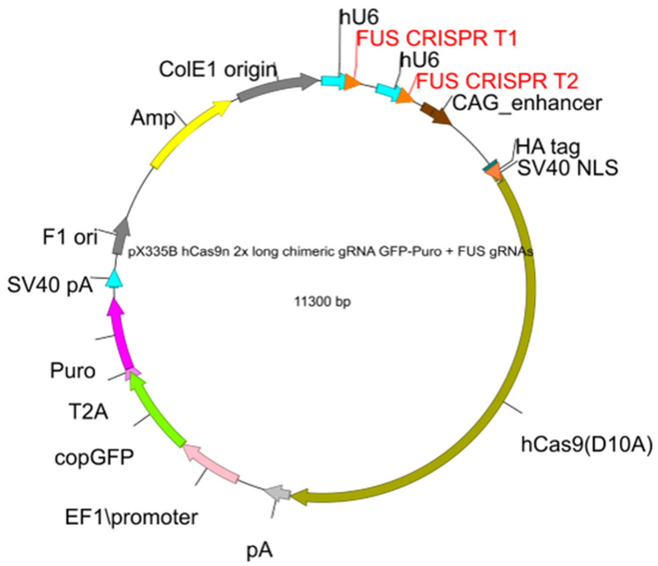

Figure S1: Plasmids used in this study

px335B hCas9n 2x long chimeric gRNA GFP-Puro + FUS gRNAs is expressing the nikase Cas9n and both gRNAs for the double nicking approach. pEX-K4 FUS-eGFP correction plasmid acts as an HDR template containing both up- and downstream homology arms, a linker containing a TEV site, S-peptide, PreScission Site and eGFP.

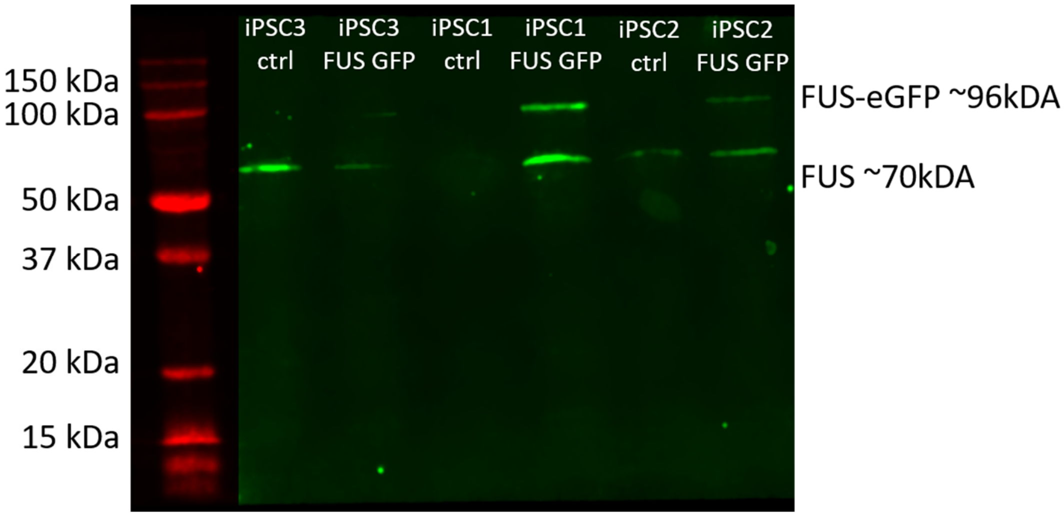

Figure S2: Westernblot after CRISPR mediated genome engineering
All untreated iPSC line show no FUS-eGFP fusion protein. After successful CRISPR FUS-eGFP is detectable in the Westernblot.

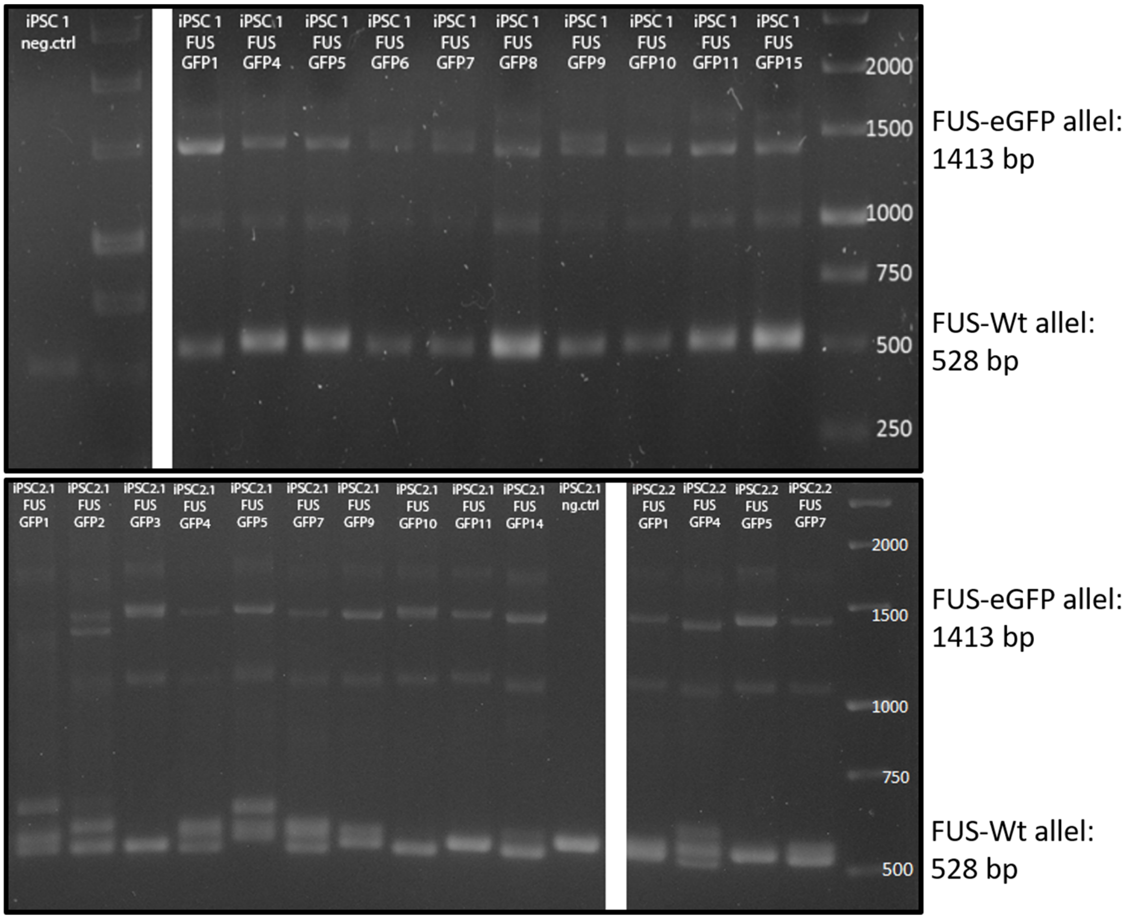

Figure S3: Genotyping PCR results of iPSC1 and iPSC2 lines
After successful CRISPR a eGFP tagged allel with the size of 1413bp can be observed. The FUS-wt allel is 528 bp and can be seen in the unCRISPRed controls as well as the heterozygous clones. Clones iPSC1 FUS-GFP 5, iPSC2.1 FUS-GFP 5 and iPSC2.2 FUS-GFP 7 have been used in this study.

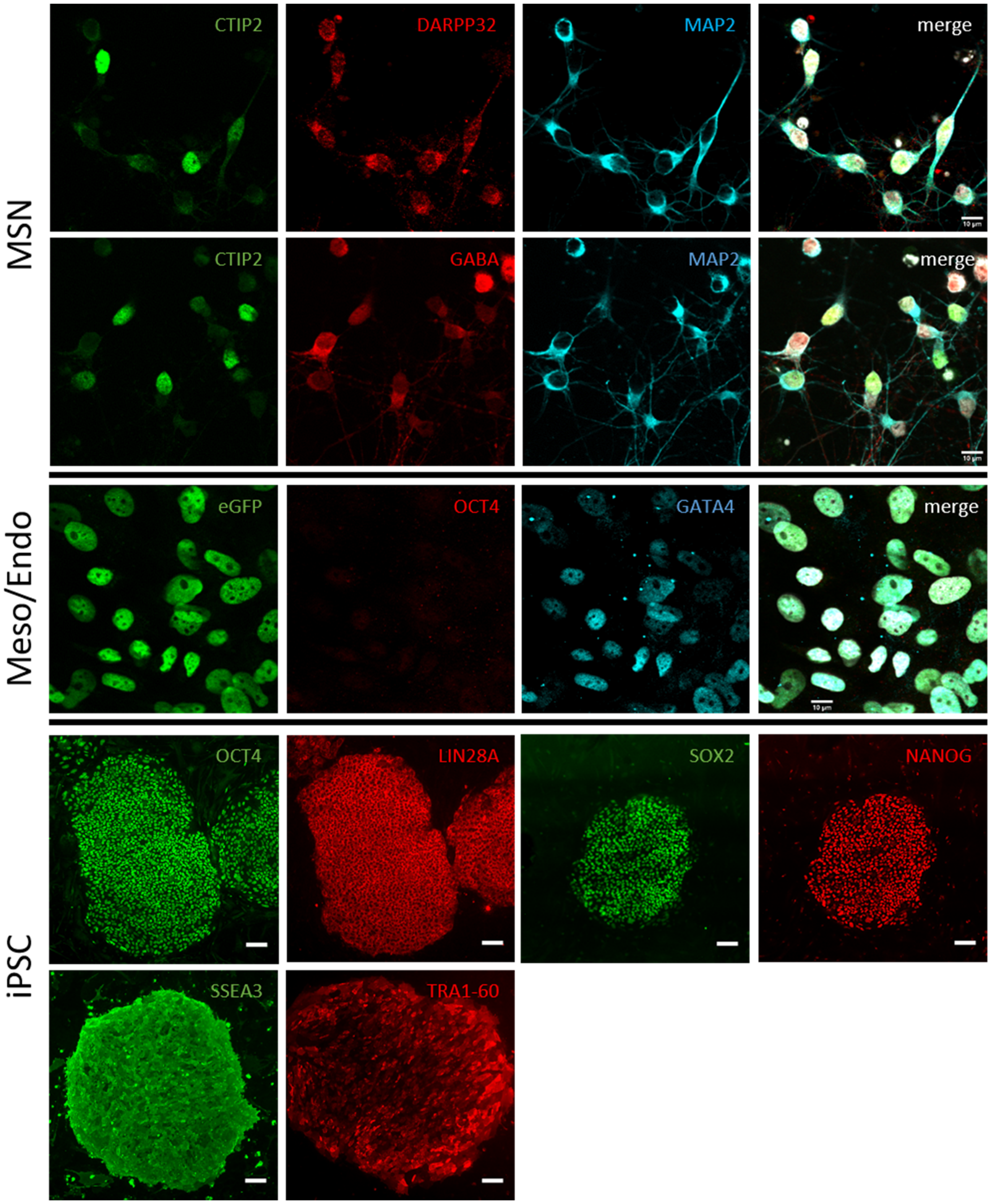

Figure S4: Representative immunofluorescence stainings of the used differentiation models
MSN show expression of hallmarks DARPP32, CTIP2 and GABA. Cells differentiated in Meso- / Endodermal linage lost OCT4 expression and are positive for GATA4.Bonafide iPSC were characterized by positive staining of OCT4, LIN28A, SOX2, NANOG, SSEA3 and TRA1-60. Scale bar for MSN an Meso/Endo represents 10 µm; Scale bar for iPSC represents 200 µm.
